# Prescription of medication before and after acute admission with nonspecific abdominal pain

**DOI:** 10.1186/1757-7241-23-S1-A23

**Published:** 2015-07-16

**Authors:** Lena Boulakh, Morten Lamberts, Hanne Jørsboe

**Affiliations:** 1 Akutafdelingen, Nykøbing F. Sygehus, Nykøbing Falster, Denmark; 2 Kardiologisk Afdeling, Gentofte Sygehus, Gentofte, Denmark

## Background

Abdominal pain is among the most common symptoms leading to referral to emergency departments in Denmark. Many of these patients are discharged without a specific diagnosis despite thorough work-up. We set out to assess the drugs prescribed to these patients in order to identify preconditions prior to admission based on their pharmaceutical treatment. This aims to generate hypotheses of admission causes derived from the drugs the patients have been prescribed since knowledge of drugs consumption patterns can improve treatment regimes.

## Methods

National databases of patient registry (ICD-10 code R10) and prescriptions were used to identify use of medication in patients (age 16-100) admitted to hospital with abdominal pain in 2010. Exclusion criteria were surgical procedure work-up or diagnosis of gastrointestinal, renal, or gynecological disease one year prior to admission. Medical use at two time points was recorded: 90 days prior to admission and 90 days after discharge. New users were defined as no prescription of the same drug 90 days prior to admission. Particular attention was made to antibiotics, oral analgesics, and antacids. Data is presented as numbers and percentages.

## Results

Nationwide, 12,081 patients were discharged with a ICD-10 code of "acute abdominal pain" (64.4% women, 35.6% men). Prior to admission, the percentage of patients using the symptomatic medications was: Oral analgesics 16.1% and antacids 21.2%. After discharge there was a significant number of new users of: oral analgesics 48.5%, especially NSAIDs and opioids, and antacids 47.2%. Of special interest is that patients were prescribed antibiotics at both times; before 19.5% and after 63.8% (p < 0.05).

## Conclusion

A substantial proportion of the patients collected a prescription of antibiotics, painkillers, and antacids. Further investigations of chart reviews and knowledge of the processes of clinical decision-making are needed to establish if it is an unrestricted prescription policy rather than an appropriate work-up. Additional studies of side effects and prescription profiles could provide insights to the symptoms leading to admission.

